# Tuning the Electronic Property of Reconstructed Atomic Ni‐CuO Cluster Supported on N/O‐C for Electrocatalytic Oxygen Evolution

**DOI:** 10.1002/advs.202310181

**Published:** 2024-03-21

**Authors:** Xinran Li, Yang‐Yi Liu, Cheng Li, Huaiguo Xue, Songqing Chen, Qiang Xu, Huan Pang

**Affiliations:** ^1^ School of Chemistry and Chemical Engineering Yangzhou University Yangzhou Jiangsu 225009 P. R. China; ^2^ Shenzhen Key Laboratory of Micro/Nano‐Porous Functional Materials (SKLPM) Academy for Advanced Interdisciplinary Studies SUSTech‐Kyoto University Advanced Energy Materials Joint Innovation Laboratory (SKAEM‐JIL) and Department of Chemistry Southern University of Science and Technology (SUSTech) Shenzhen Guangdong 518055 P. R. China; ^3^ School of Electrical Engineering Engineering Technology Research Center of Optoelectronic Technology Appliance Tongling University Tongling Anhui 244061 P. R. China; ^4^ Hefei Comprehensive National Science Center (Anhui Energy Laboratory) Hefei Anhui 230051 P. R. China

**Keywords:** electrocatalysis, nanoalloy, pre‐catalyst, single atom

## Abstract

Electrochemical activation usually accompanies in situ atom rearrangement forming new catalytic sites with higher activity due to reconstructed atomic clusters or amorphous phases with abundant dangling bonds, vacancies, and defects. By harnessing the pre‐catalytic process of reconstruction, a multilevel structure of CuNi alloy nanoparticles encapsulated in N‐doped carbon (CuNi nanoalloy@N/C) transforms into a highly active compound of Ni‐doped CuO nanocluster supported on (N/O‐C) co‐doped C. Both the exposure of accessible active sites and the activity of individual active sites are greatly improved after the pre‐catalytic reconstruction. Manipulating the Cu/Ni ratios of CuNi nanoalloy@N/C can tailor the electronic property and d‐band center of the high‐active compound, which greatly optimizes the energetics of oxygen evolution reaction (OER) intermediates. This interplay among Cu, Ni, C, N, and O modifies the interface, triggers the active sites, and regulates the work functions, thereby realizing a synergistic boost in OER.

## Graphical abstract

By harnessing the pre‐catalytic process of reconstruction, a multilevel structure of CuNi alloy nanoparticles encapsulated in N‐doped carbon transforms into a high‐active compound of Ni‐doped CuO nanocluster support on N/O co‐doped C. Manipulating the Cu/Ni ratios can tailor the electronic property and d‐band center of the high‐active compound, which greatly optimizes the energetics of oxygen evolution reaction intermediates.

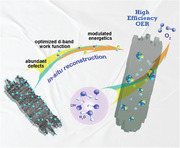

## 1 Introduction

The oxygen evolution reaction (OER) is a critical process in various advanced energy conversion devices such as water electrolysis and rechargeable metal‐air batteries, in which process involves a four‐electron transfer and several adsorbed intermediates (OH*, O*, and OOH*).^[^ ^1^, ^2^ ^]^ However, the kinetics of O─O bond formation and the tri‐phase reaction at the interface of gas‐diffusion and ion adsorption lead to sluggish rates, which require electrocatalysts to provide abundant active sites to facilitate OER.^[^ ^3^, ^4^ ^]^ Comparing to the benchmark of noble‐based electrocatalysts, transition metal compounds, with the superiority of earth‐abundance, non‐toxicity, and regulable electronic properties, serve as promising candidates for long‐lived and high‐efficiency OER catalysts.^[^ ^5^, ^6^ ^]^ Nevertheless, most transition metal compounds require relatively high overpotentials to drive OER efficiently, resulting in increased energy consumption.^[^ ^7^, ^8^ ^]^ Therefore, designing and fine‐tuning the electronic properties of the OER catalysts to decrease overpotentials is necessary.^[^ ^9^, ^10^ ^]^

The copper‐based materials are one of the most widely used materials for electrochemistry and catalysis.^[^ ^11^ ^]^ However, when reported as OER electrocatalyst, copper‐based materials typically show less satisfactory catalytic performance compared to the well‐studied nickel/cobalt‐based composites.^[^ ^12^ ^]^ This is because the filling of the anti‐bonding state of Cu (3d^10^ 4s^1^) is higher than those of Ni (3d^8^ 4s^2^) and Co (3d^7^ 4s^2^) when O 2p band of oxygen intermediates hybridizes with the metal d band.^[^ ^13^ ^]^ Through isolating active sites can realize the regulation of local coordination environment so as to achieve active and selectivity.^[^ ^14^ ^]^ Atomic clusters contain diverse geometric and electronic structures, plentiful active sites, and more moderate interaction intensity as well as overcome the problem of unsatisfactory stability of the single atom.^[^ ^15^ ^]^ Moreover, with the electrochemical conditions of OER in alkaline solution, most transition metal‐based catalysts tend to undergo interfacial evolution and activated reconstruction.^[^ ^16^ ^]^ The most essential driving force for reconstruction is the surface chemical conversion. It has been found that the adaptive surface evolution and degree of reconstruction are initially determined by the physicochemical features of pre‐catalysts, and then strongly impacted by the reaction and service conditions as well as their interactions during OER, such as local pH and its gradient distribution, applied potential, types and concentration of exotic ions, and external fields on top of the catalysts.^[^ ^17^ ^]^ The reconstruction can be either reversible or irreversible over time based on intrinsic properties of pre‐catalysts and external conditions.^[^ ^18^ ^]^ The process accompanying atomic rearrangements forms an active amorphous phase or atomic nanocluster/single atoms with abundant dangling bonds, vacancies, and defects, which enhances the exposure of accessible active sites and activity of individual active sites.^[^ ^19^ ^]^

Herein, a series of CuNi alloy nanoparticles encapsulated in N‐doped carbon (noted as CuNi nanoalloy@N/C) were obtained by carbonizing Cu/Ni bi‐metal complexes. When employed as OER pre‐catalysts, the CuNi nanoalloy@N/C undergoes an in situ reconstruct process transforming into a high‐active compound of Ni‐CuO clusters supported on N/O co‐doped C. Controlling the Cu/Ni ratios within CuNi nanoalloy@N/C can tune the electronic property and d‐band center of the high‐active compound, greatly optimizing the adsorption/ desorption strength of intermediates. The interplay among the Cu, Ni, C, N, and O modifies the interface and triggers on the active sites as well as regulates the work functions, thus synergistically boosting an active OER.

## 2 Results and Discussion

**Figure 1:**
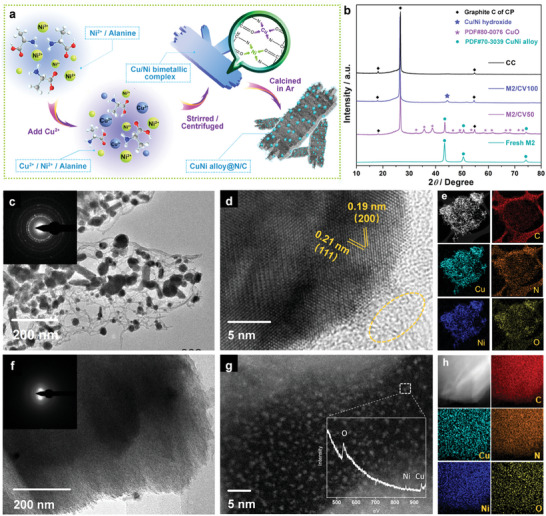
a) Schematic illustration for the synthesis process of CuNi nanoalloy@N/C. b) XRD patterns of fresh M2 and M2‐CV. c,d) TEM images of M2, and the insets of c) Selected‐area electron diffraction (SAED) patterns; e) corresponding TEM‐EDS mapping images. Structural characterization of reconstructed M2 after CV activation for 100 cycles: f) TEM images and the corresponding SAED patterns; g) the AC‐HAADF‐STEM image and the high‐loss EELS image of corresponding Cu/Ni oxide cluster; h) corresponding TEM‐EDS mapping images.

CuNi nanoalloy@N/C was prepared via a two‐step synthetic route. The synthetic route was reasonably designed, as depicted in **Figure** **1**a, with Ni (II) initially coordinating with alanine, followed by adding a high‐concentration Cu (II) solution for further coordination and substitution reactions. This sequence was chosen because Cu (II) ion is more prone to forming stable coordination compounds compared with Ni (II) ion due to its smaller ion radius and higher ionization potential. (More detailed information can be supplied in The Experimental Section). The Cu/Ni bimetallic complexes (P1–P7) with varying Cu/Ni ratios were formed as a result and Table S1 (Supporting Information) provides specific details about the amounts of reagents used in the synthesis. Figure S1 (Supporting Information) shows the optical photographs of Cu/Ni bimetallic complexes, in which can be observed that the color of P1–P7 changes slightly with the varying Cu/Ni ratios. Field emission scanning electron microscopy (FE‐SEM) images in Figure S2 (Supporting Information) reveal that the complexes exhibit a 2D plate morphology with a micro‐scale. Additionally, the samples exhibit a gradual morphology transformation that the length‐width ratio was enhanced when decreasing the Cu/Ni molar ratio.

Thermogravimetric (TG) analysis was conducted on P1–P7 calcined in an Ar atmosphere. The significant weight loss (≈70%) at 200–300 °C is related to the decomposition of P1–P7, as shown in Figure S3 (Supporting Information). The platforms in the TG curves indicate fractures in the different coordination bonds, demonstrating the effect of Cu/Ni ratio on the stabilities of complexes. The increase in temperature leads to a collapse in the coordination structures of P1–P7, releasing partial C, N, H, and O as gas/ion currents. This process causes the formation of an atomically dispersed porous structure of N‐doped amorphous carbon‐supported CuNi alloy nanoparticles.

The X‐ray diffraction (XRD) patterns of P1–P7 in Figure S4a (Supporting Information) show typical peaks at ≈10°, possibly due to the 2D plate crystalline with specific crystallographic plane orientation. Additionally, Figure S4b (Supporting Information) exhibits the XRD patterns and reveals characteristic peaks at 43.5°, 50.6°, and 74.1°, which can be well indexed to the (111), (200), and (220) lattice planes of the CuNi alloy (JCPDS#70‐3039). The slight shift of characteristic peaks with the increasing Ni proportion indicates the incorporation of Ni atoms into the lattice substituting Cu atoms.^[^ ^20^ ^]^ The P1–P7 precursors retained their 2D morphology with a micrometer size after calcination, as shown in Figure S5 (Supporting Information). The size of the samples decreased as the Ni proportion increased, and their morphologies evolved from microplates to curly nanobelts. The FT‐IR spectra of P1–P7 and M1–M7 are shown in Figure S6 (Supporting Information)., in which the stretching modes of carboxylate, C─C, and C─H bending vibrations were observed to peak within the spectral region of 1650–1250 cm^−1^. The vibrational bands at 3455, 3261, 1621, and 1283 cm^−1^, which correspond to ν(O─H), ν(N─H), free water, and δ(C─H) respectively, are the characteristic peaks of crystal water complexes.^[^ ^21^ ^]^ The FT‐IR spectra of M1–M7 (Figure S6b, Supporting Information) display peaks corresponding to O─H, C═O, C─O, and C─O─C groups, respectively. These peaks may be attributed to a small number of functional groups or residual dangling bonds after the calcination process, resulting from the breakage of the intact coordination number.^[^ ^22^ ^]^ During the electrochemical reaction, active sites of carbon‐based materials mainly reside within the lattice defects or edges generated by the break or redistribution of charge/spin in the sp_2_ conjugated within the carbon matrix.

The as‐obtained pre‐catalysts undergo an in situ reconstruction process during electrochemical activation of circulation voltammetry (CV), and the morphological and structural change of M2 are demonstrated in Figure 1. Figure 1b shows the XRD patterns of fresh M2 and M2 after CV. When the CV is performed for 50 cycles, the XRD pattern of M2‐CV50 shows the combined characteristic peaks of CuNi alloy (JCPDS#70‐3039) and CuO (JCPDS#80‐0076). These characteristic peaks of M2‐CV50 vanish after 100 cycles of CV, with only an ambiguous peak at ≈44.3°, speculated to be caused by the formation of a CuNi hydroxide. The FE‐SEM and Transmission electron microscopy (TEM) images of M1‐7 are presented in Figure S6 (Supporting Information) and Figure 1c,d. These images illustrate that the CuNi alloy NPs have a size of approximately 30 nm and are surrounded by amorphous carbon. Figure 1d indicates the presence of lattice fringes with dimensions of 0.21 and 0.19 nm, which can be assigned to the (111) and (200) crystal faces of the CuNi alloy, respectively. The selected area electron diffraction (SAED) pattern inserted in Figure 1c suggests the polycrystalline nature of the sample due to the presence of diffraction rings. Furthermore, Figure 1d highlights the presence of functionalized carbon produced during the pyrolysis process, which is shown as a yellow circle. The EDS mapping images (Figure 1f) and EDS line scan in Figures S7–S11 (Supporting Information) show that the atomic homogeneous CuNi alloy nanoparticles are evenly distributed throughout the hierarchically structured samples and mainly coated with N‐doped carbon.

Figure S12 (Supporting Information) exhibits the TEM images of M2 after CV test of 50 and 100 cycles, respectively. It can be speculated that as the CV test went on, CuNi alloy nanoparticles gradually transformed into 2D sheet Cu/Ni hydroxide with slight agglomeration of the sample. A strong metal‐support interaction (SMSI) which is rigorously defined as the interaction between metal and support could impact metal dispersion as well as alter the electronic and geometric structures of loaded metals. The dispersed metals typically exhibit a highly dispersed form either as layer‐like structures or few‐atom per single‐atom clusters because of the electronic metal‐support interaction or reactive metal‐support interaction.^[^ ^18^ ^]^ This means Cu and Ni sites tend to be evenly distributed to form oxide clusters, only a small number of single atoms are distributed on carbon, as shown in Figure 1g. According to the content of each element of M2 in Figure S13 (Supporting Information), Cu 53.67%, Ni 12.49%, C 25.07%, N 7.01%, O 1.76%. It can be speculated that after the pre‐catalytic transform from CuNi nanoalloy‐N/C to Ni‐doped CuO nanocluster‐N/C, the content of O increased significantly, while the content of C and N partially decreased. Single atoms are supported on the N‐doped C which shows a thermodynamic instability above 0.207 V theoretically, while OER generally occurs at a high potential over 1.4 V (vs RHE). This means that carbon may show the unsatisfied stability to support the single atom during OER.^[^ ^23^ ^]^ Based on this, the content of single atom is very small in the reconstructed M2, and it cannot continuously catalyze OER due to the instability of carbon framework. Therefore, the oxide clusters playing a major role in the reaction process should be mainly analyzed.^[^ ^24^ ^]^ The aberration‐corrected high‐angle annular dark‐field scanning transmission electron microscope (AC‐HAADF‐STEM) measurement with sub‐angstrom resolution is used to confirm the presence of Ni /Cu oxide cluster (marked in yellow) immobilized on CNO frameworks, as shown in Figure 1g. The EDS mapping images of M2‐CV in Figure 1h prove the uniform distribution of all atoms at micron‐scale.

X‐ray photoelectron spectra (XPS) were conducted to provide evidence for the changes in electronic properties of the M1–M7 as shown in Figure S14 (Supporting Information). The Cu 2p_1/2_ (≈951 eV) and Cu 2p3/2 (≈932 eV) peaks in Figure S14a (Supporting Information), the slight peak shift indicates the different oxidation states of M1–M7. Similarly, in Figure S14b (Supporting Information), the shift of peaks in Ni 2p_1/2_ (≈868 eV) and Ni 2p_3/2_ (≈852 eV) reveal the various oxidation of metallic states of Ni^2+^ and Ni^0^. Notably, as the proportion of Ni increases from M1 to M7, the Cu 2p binding energy slightly decreases while the Ni 2p binding energy increases. This trend can be explained by the redistribution and disorder of electrons in the coordination environment of the atoms, which leads to an opposite shift of the Cu 3d band towards the Fermi level and subsequently tunes the adsorption energies of OH^*^.^[^ ^23^ ^]^ Furthermore, the M‐O peaks of M1 and M7 in the O 1s spectrum (Figure S14c, Supporting Information) reveal that monometallic composites (M1 with Cu and M7 with Ni) exhibit exacerbated oxidation compared to bimetallic composites (M2–M5). This difference may be attributed to the formation of a “metallic protection” by the CuNi alloy, which inactivates the oxidation of the metallic materials. Moreover, the N 1s and C 1s spectra of M1–M7, shown in Figure S14d,e (Supporting Information), provide further insights. The N 1s spectrum displays two peaks corresponding to graphitic N (401.8 eV) and pyridinic N (398.2 eV). The C 1s spectrum consists of peaks representing the C─C sp^2^ bond at 284.5 eV, C─C sp^3^ bond at 285.3 eV, and C─N bond at 286.2 eV. Finally, the shifts in peak centers observed in the N 1s and C 1s spectra of M1–M7 illustrate the changes in the content of N or C species, possibly resulting from the different electronic affinities of Cu and Ni in the CuNi alloy with different Cu/Ni ratios. The samples' porous feature is evident from the N_2_ adsorption‐desorption isotherms (Figure S15, Supporting Information). After calcination, the Brunauer‐Emmett‐Teller (BET) surface area of CuNi alloy@ N/C increased nearly tenfold compared to Cu/Ni bimetallic complexes. Notably, M2 has the highest BET surface area (198.24 m^2^ g^−1^) among M1–M7. The porous structure provides abundant exposed active sites and promotes electron transfer and mass transport during electrochemistry reactions.

**Figure 2:**
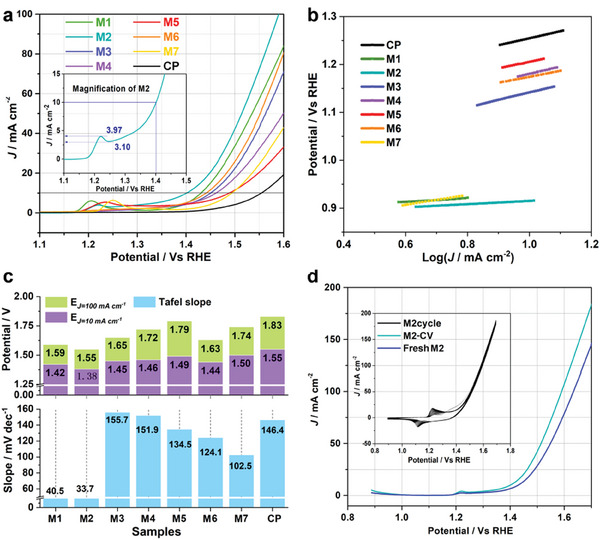
a) Linear scan voltammetry (LSV) curves of the pre‐catalysts after electrochemical activation of 100‐cycle circulation voltammetry (CV) for M1‐M7; b) the corresponding Tafel slope with an overpotential at ≈10 mA cm^−2^ of M1‐M7; c) Comparison of the values of Tafel slope with potentials at 10 mA cm^−2^ (E*_J_*_= 10 mA cm_^−2^) and 100 mA cm^−2^ (E*_J_*_= 100 mA cm_^−2^) of M1–M7 after electrochemical activation; d) LSV curves of fresh M2 and M2 after 100‐cycle CV test, and the inset in d) the corresponding CV curves.

The electrochemical activation of pre‐catalysts is performed by CV test (Figure S16, Supporting Information; **Figure** **2**d), all of which undergo the reconstruction process and show lower overpotential after the in situ reconstruction. In Figure 2d, after the activation of CV, the REDOX peak of M2 decreases and shifts, which is caused by the oxidation and reconstruction of CuNi alloy NPs. The linear scan voltammetry (LSV) curves of after the pre‐catalytic procedure are shown in Figure 2a, M2 exhibits an earlier current response compared to the other samples, with only a potential of 1.41 V (overpotential of 180 mV) to achieve a current density of 10 mA cm^−2^. Furthermore, a potential of 1.59 V (overpotential of 360 mV) is needed to reach a current density of 100 mA cm^−2^. The anodic peaks observed in the polarization (Insert of Figure 2a) are attributed to oxidation of the metal sites occurring on the surface. Tafel slopes of M1–M7 electrochemical activation of CV were obtained through tests and post‐calculation of LSV, as shown in Figure 2b. In Figure 2c, overpotentials and Tafel slopes of M1–M7 after CV activation are compared, M2 shows the lowest overpotential and Tafel slope of the catalysts, exhibiting a first‐class electrocatalytic performance. The electrochemically active surface area (ECSA) and surface roughness were calculated using the electrochemical double‐layer capacitance (C_dl_) in the non‐faraday range, as represented in Figure S17 (Supporting Information). After electrochemical activation, M2‐CV exhibited the highest C_dl_ value of 22.51 mF cm^−2^, indicating significant roughness and a substantial number of active sites. Analyses of the intrinsic kinetics changes were conducted through the three‐dimensional AC impedance curves of M1–7, wherein the Cu/Ni ratio was regulated (Figure S18, Supporting Information). A lower charge‐transfer resistance (R_ct_) is favorable as it indicates a lower interface reaction resistance and better OER performance. The structure, conductivity, degree of wettability, and access of the catalyst to the electrolyte greatly influence the reaction rate of the working electrodes/substrates.

Raman spectra (Figure S19, Supporting Information) were conducted to show the prominent features of the functionalized carbon in M1–M7. The D band (≈1339 cm^−1^) denotes the defect sites of disordered sp^3^ carbons, while the G band (≈1594 cm^−1^) reflects the sp^2^ graphitic carbons. The I_D_/I_G_ ratios of the samples, ranging from 0.81 to 1.02, are affected by the amount of Cu and Ni present. The higher the I_D_/I_G_ ratios, the more the electron transfer and catalytic centers improve. Notably, M2 generated a relatively high I_D_/I_G_ ratio, implying a proper metallic ratio that induces more defects to the catalyst surface. Previous literatures has reported that the carbon renders large surface area enhances the ability of electrolyte diffusion, smooths the pathway for electron and mass transfer, and stabilizes the skeletal structure. The adjustment of Cu/Ni ratio results in modulated local coordination environments and electronic structures through the synergistic interplay between Cu and Ni. This generates defects at lattice or edges, forming abundant active sites that ultimately facilitate the OER.

**Figure 3:**
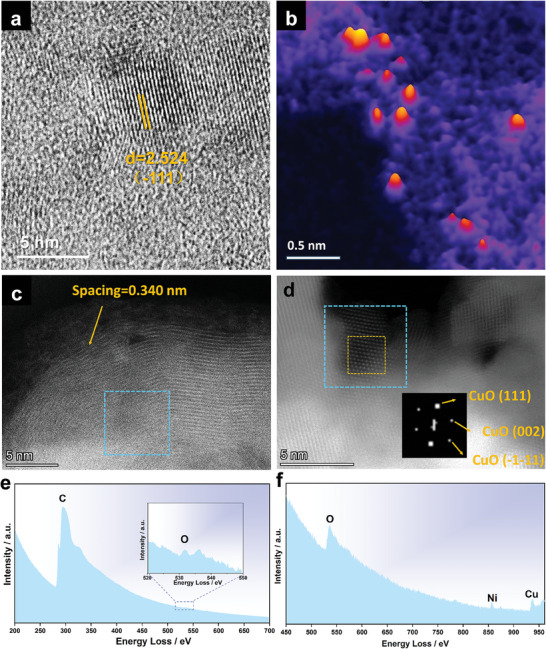
a) HR‐TEM of M2‐CV; b) The corresponding 3D false‐color image of one HAADF‐STEM image of M2‐CV, in which different colors represent the intensities of pixels, (The red peaks in the 3D image indicate the precise locations of individual metal atoms); e,f) High‐loss EELS images and the corresponding selected area (marked with blue square) of c,d).

To investigate the process of in situ reconstruction of M2, the structural characterization and valence states after electrochemical activation were obtained through the characterization of high‐resolution transmission electron microscopy (HRTEM), XPS, and AC‐HAADF‐STEM. The HRTEM images in **Figure** **3**a depict the obvious reconstruction of M2 after CV activation for 100 cycles (M2‐CV). The 3D CuNi alloy NPs@N/C gradually converted into 2D hetero‐plate of Ni‐doped CuO cluster supported on CNO frameworks. The planes of (−111) of CuO (JCPDS#80‐0076) in Figure 3a illustrate the surface evolution and structure reconstruction during the electrochemical activation. The application of a 3D false‐color image in Figure 3b effectively demonstrates the presence of single metal atoms on the amorphous support within the M2‐CV. Figure 3e,f exhibits the electron energy loss spectrum (EELS) and the corresponding selected area is shown in Figure 3c,d (blue square), which further confirms the composition of M2‐CV at the atomic level. After the reconstruction, the catalyst converts into a hetero‐plate of Ni‐doped CuO cluster (Figure 3d,f) supported on CNO frameworks (Figure 3c,e).

**Figure 4:**
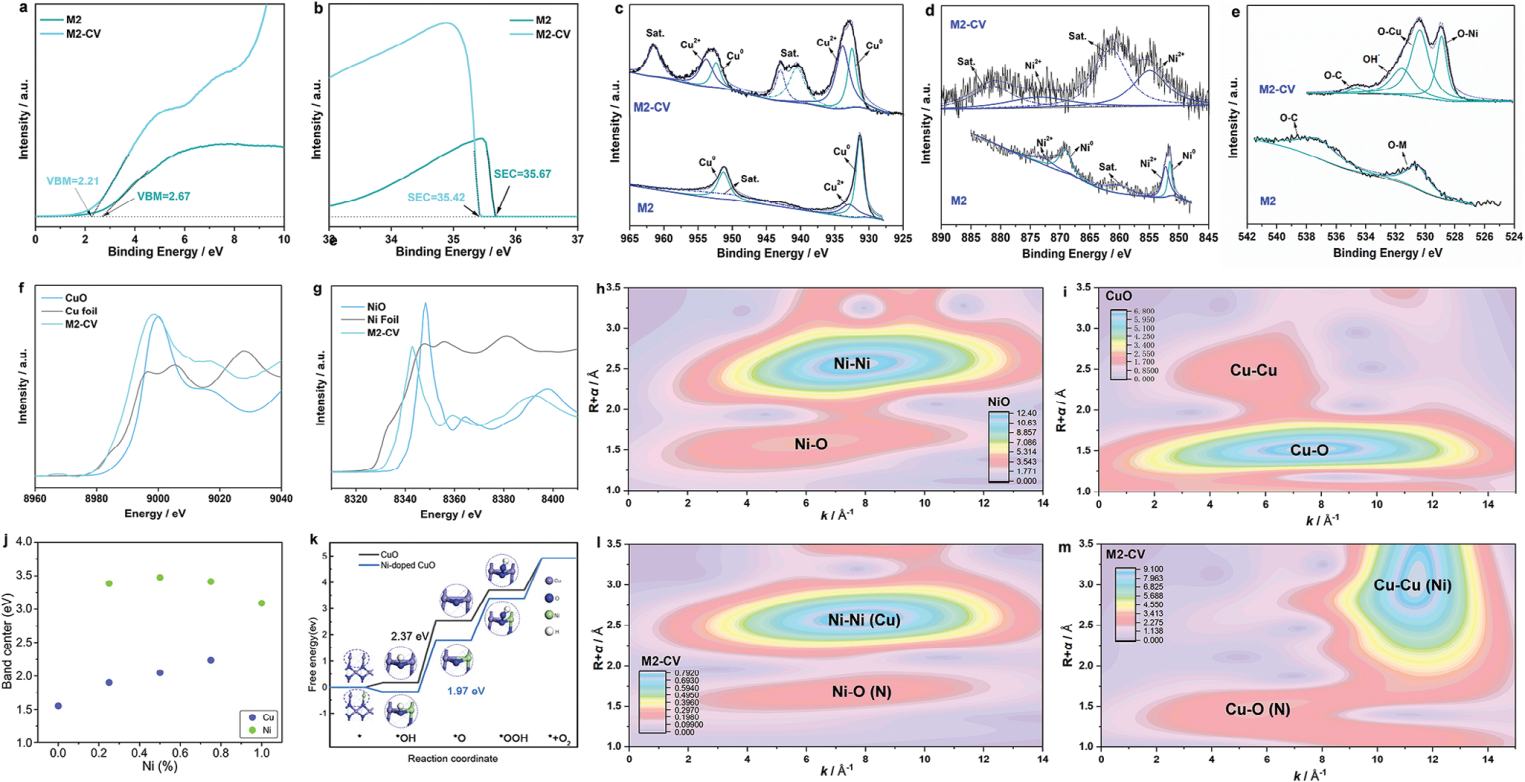
Ultraviolet Photoelectron Spectra (UPS) of M2 and M2‐CV: a) valence‐band spectra and b) secondary electron cutoff. The high‐resolution XPS spectrums of M2 and M2‐CV: c) Cu 2p, d) Ni 2p, e) O 1s. f) normalized Cu K‐edge XANES spectra, g) normalized Ni K‐edge XANES spectra, Wavelet Transform spectra WT‐EXAFS contour plots of h) commercial NiO, i) commercial CuO, l) metallic Ni, and m) Cu respectively; j) d‐band centers of Cu‐Ni systems, k) Free energy profile of OER process on Cu(111) and Ni‐doped Cu(111) surfaces. The structures of key intermediates are shown in the insets. The barriers of PDS are also marked.

Ultraviolet photoemission spectroscopy (UPS) is used to explore the electronic properties of reconstructed pre‐catalysts in depth. As shown in **Figure** **4**a, the valence band (*E_V_*) for M2 and M2‐CV is located at 2.21 and 2.45 eV below Fermi energy (*E_F_*) by linearly extrapolating the leading edge of the spectrum to the baseline. In addition, the work function (*Ф*) can be calculated using *Ф* = *hv*–*E*_onset_ where *h* is the incident photon energy (40.2 eV) and *E*_onset_ is the onset level related to the secondary electrons, as shown in Figure 4b. The corresponding work functions of M2 and M2‐CV are calculated to be 4.78 and 4.53 eV, which reflects the dynamics of electrons on the surface of the samples. The M2‐CV shows higher Fermi level and smaller work function than M2, which illustrates that the pre‐catalyst possesses better electrical conductivity and could deliver electrons more easily after the in situ reconstruction. All the characterizations reveal a significant reconstruction during pre‐catalytic process involving the oxidation of the metal, formation of oxygen/nitrogen‐related defect sites, and the absorption of carboxylate groups on the catalyst interface.^[^ ^25^ ^]^

In Figure 4c, XPS of Cu 2p for M2‐CV at ≈953.5 and ≈932.8 eV indicate the main metallic state of Cu^2+^ and Cu^0^ respectively. And the main metallic state of Ni covert into content of Ni^2+^ in M2‐CV, in line with the O 1s spectrum in Figure 4e (OH^−^ 531.6 eV, O─Cu 530.4 eV, and O─Ni 528.9 eV).^[^ ^26^ ^]^ In Figure S20a,b (Supporting Information), the characteristic peaks of C 1s and N 1s become more complicated after the electrochemical reaction due to the new appearance of new C═C (283.5 eV), C─O (287.3 eV), C─F bonds from Nafion. The N 1s shows that graphitic N (401.8 eV) fades away and the M─N (399.5 eV) comes out during the OER. In Figure S21 (Supporting Information), the Mott‐Schottky curve slope of M2 increases after activation indicating a higher carrier concentration, which is possibly due to the introduction of electrons or holes, enhancing their mobility. This leads to greater availability of electrons and holes for charge transport.

The X‐ray absorption near edge structure (XANES) spectra of Cu K‐edge and Ni K‐edge are exhibited in Figure 4f,g. The absorption energy of M2‐CV is between those of Cu foil and commercial CuO as well as Ni foil and commercial NiO suggesting that both Cu and Ni in M2‐CV have a valence state between 0 and +2. Wavelet Transform Spectra WT‐EXAFS contour plots of the Cu and Ni K edge‐extended X‐ray absorption fine structure (FT‐EXAFS) of M2‐CV and commercial NiO/CuO are shown in Figure 4h–m respectively. Figure 4h shows two prominent peaks at ≈1.60 and 2.60 Å, corresponding to the nearest neighbor Ni─O and next‐nearest neighbor Ni─Ni coordination shells in commercial NiO.^[^ ^27^ ^]^ While in Figure 4l, the peak attributed to Ni─O (N) interaction located at 1.75 Å and Ni─Ni(Cu) is shifted to 2.75 Å (Figure S22, Supporting Information). Figure 4i shows two prominent peaks at ≈1.55 and 2.55 Å, corresponding to the nearest neighbor Cu─O and next‐nearest neighbor Cu─Cu coordination shells in commercial CuO. In Figure 4m that the Fourier transformed Cu K‐edge EXAFS of M2‐CV, the peak belonging to Cu─O (N) interaction located at 1.50 Å and Cu─Cu (Ni) is shifted to 3.05 Å (Figure S23, Supporting Information). The details structural parameters calculated from the EXAFs fittings are shown in Table S2 (Supporting Information), comparing to the commercial CuO and NiO, these changes of distance between Cu─O/Ni─O atoms and the coordination number of Cu/Ni─O in M2‐CV are mainly due to the Cu and Ni sites with an oxidation state are arranged in a the unique heterostructure of lattice structure of Ni‐doped CuO immobilized on CNO frameworks.

The density functional theory (DFT) calculation was further performed to understand the superior performance of CuNi alloy pre‐catalysts on OER. The electronic structures of the bulk alloy systems are first studied. As shown in Figure 4j, the d‐band center of Cu upshifts through interaction with Ni, resulting in increased oxygen adsorption of the alloy surface. Then we simulated the OER process on the oxidized surfaces. Compared with the widely studied NiOOH, CuO is commonly considered as an inefficient electrocatalyst. In bulk, the coordination number of Cu is 4. As exposed on the surface, half of the copper atoms are undercoordinated with coordination number of 3 which are supposed to be the reactive sites. In the M2‐CV, the CuO nanocluster with Ni doped shows coordination numbers of 2.4/2.4 with Cu─O /Cu─N respectively (Table S2, Supporting Information).^[^ ^28^ ^]^ As shown in Figure 4k, the active Cu sites are readily occupied to form *OH, corresponding to Cu^2+^ species. However, the further oxidation to *O is quite difficult since copper has been in its highest valence oxidation state. As a result, the formation of *O from *OH is the potential determining step (PDS) with an energy change of 2.37 eV. After introducing under‐coordinated nickel, the conversion between Ni^2+^ and Ni^3+^ may stabilize *O intermediate and thus significantly promote the oxidation step to *O. The barrier of PDS decreases to 1.97 eV. Therefore, the CuO surface with Ni dopant exhibits improved performance on electro‐catalyzing OER.^[^ ^29^ ^]^

## 3 Conclusion

Taking advantage of the electrochemical activation of in situ atom rearrangement, the pre‐catalysts transformed from the original multilevel structure of CuNi alloy nanoparticles encapsulated in N‐doped carbon (CuNi nanoalloy@N/C) into a high‐active compound of Ni‐doped CuO cluster supported on CNO frameworks. Through manipulating the Cu/Ni ratios of CuNi nanoalloy@N/C, the electronic property and d‐band center of the high‐active compound are tailored to modulate the adsorption/desorption energy of OER intermediates. This optimized interplay within Cu, Ni, C, N, and O modifies the interface, triggers the active phases, and regulates the work functions of active sites, thereby realizing a synergistic boost in OER. Utilizing SMSI effects induced by electrochemistry activation is a promising approach to enhance metal dispersion and create metal/support interfaces on supported metal catalysts. Eventually, exquisite control of SMSI can be realized after properly exploiting the dynamic transformation processes, which helps with rational design of multicomponent catalysts.

## Conflict of Interest

The authors declare no conflict of interest.

## Supporting information

Supporting Information

## Data Availability

The data that support the findings of this study are available from the corresponding author upon reasonable request.
