# Supplementary material for: Tuning the Electronic Property of Reconstructed Atomic Ni‐CuO Cluster Supported on N/O‐C for Electrocatalytic Oxygen Evolution
Source: Adv Sci (Weinh). 2024 Mar 21;11(22):2310181. doi: 10.1002/advs.202310181 (PMC11165517; doi:10.1002/advs.202310181)
Supplement: Supplementary file 1 — Supporting Information [file ADVS-11-2310181-s001.pdf]

ADVANCED SCIENCE
Open Access

Supporting Information

for *Adv. Sci.*, DOI 10.1002/advs.202310181

Tuning the Electronic Property of Reconstructed Atomic Ni-CuO Cluster Supported on N/O-C for Electrocatalytic Oxygen Evolution

*Xinran Li, Yang-Yi Liu, Cheng Li, Huaiguo Xue, Songqing Chen, Qiang Xu and Huan Pang**

Supporting Information

Tuning the electronic property of reconstructed atomic Ni-CuO cluster supported on N/O-C for electrocatalytic oxygen evolution

*Xinran Li, Yang-Yi Liu, Cheng Li, Huaiguo Xue, Songqing Chen, Qiang Xu, and Huan Pang**

1

Experimental Section

Typical synthesis of the Cu/Ni bimetallic complexes: nickel acetate [$Ni(CH_3COO)_2·4H_2O$] and DL α-alanine [$C_{18}H_{23}Cl_3N_4O_4$] were mixed after adding 10 mL distilled water and stirred for 10 min until it was dissolved completely at room temperature, noted as Ni(II)-Alanine solution. Simultaneously, cupric acetate [$Cu(CH_3COO)_2\ H_2O$] was dissolved in 10.00 mL distilled water in another beaker, noted as Cu(II) solution. Then, the as-prepared Cu(II) solution was added to Ni(II) -Alanine solution and stirred for 5 min. Subsequently, 150 mL absolute ethanol was added to the solution and stirred for another 5 min, forming a Cu/Ni bimetallic complex suspension. After centrifuging the above system, the product (Cu/Ni bimetallic complexes) was obtained in air naturally. Via regulating Cu/Ni ratio, a series of Cu/Ni bimetallic complexes were synthesized and noted as P1-P7, specific amount seen in **Table S1**.

**Samples**	**P1**	**P2**	**P3**	**P4**	**P5**	**P6**	**P7**
**$Cu(CH_3COO)_2·H_2O$ /g**	0.500	0.374	0.299	0.250	0.200	0.125	0.000
**$Ni(CH_3COO)_2·4H_2O$ /g**	0.000	0.156	0.249	0.311	0.373	0.467	0.622
**DL *α*-alanine /g**	1.000	1.000	1.000	1.000	1.000	1.000	1.000
**Cu(II) solution /mL**	20.0	10.0	10.0	10.0	10.0	10.0	0
**Ni(II) solution /mL**	0	10.0	10.0	10.0	10.0	10.0	20.0
**Anhydrous ethanol/mL**	140.0	140.0	140.0	140.0	140.0	140.0	140.0
**Cu_{at}:Ni_{at}**	**1:0**	**3:1**	**3:2**	**1:1**	**2:3**	**1:3**	**0:1**

Table S1. Specific amount of reagent required to synthesize P1-P7.

Typical synthesis of CuNi nanoalloy nanoparticles @N-doped carbon (CuNi nanoalloy@N/C):

The above prepared Cu/Ni bimetallic complexes were calcined in Ar atmosphere at 600 °C. After cooling naturally at room temperature, CuNi nanoalloy@ N-doped carbon was obtained, noted as M1-M7.

Preparation of the working electrode based on carbon paper (CP):

200 µL of the above mentioned suspension were dripped onto a CP with an eara of 0.5×0.5 cm^2, which was naturally dried in the air.

After the CV test of the CuNi alloy nanoparticle@N doped C, the proportion of each element is basically invariant except for a small increase in proportion of O, of which will increase the total entropy of the compound.

Electrochemical Measurements

Measurements of OER performances of the as-prepared electrocatalysts were performed with a traditional three-electrode system (Measurements were carried out at room temperature with a CHI 760E electrochemical workstation). The working, counter and reference electrodes were a carbon paper, a graphitic rod and Hg/HgO electrode, respectively. 5.0 mg of electrocatalyst was mixed with 1.0 mL ethanol and 50 µL Nafion, and then bath sonicated for 30 min to make the catalyst ink. Then the catalyst ink was drop casted onto the working electrode of carbon paper with an area of 1 ×1 cm^2 and allowed to dry in air prior to use in the electrochemical reactions.

In-situ activation of the as-prepared pre-catalyst : It is the same as the above-mentioned three-electrode test system, the as-prepared working electrode was performed by cyclic voltammetry at the applied potentials ranging from 0.83 to 1.73 V vs. RHE for 100 cycles for the pretreatment of the working electrode of carbon paper-M1-M7. The scan rate was set at 10 mV s^{-1}.The obtained samples were finally washed with water, followed by being dried for the electrochemical measurements.

All electrochemical OER experiments were performed in 1.0 M KOH solution in an oxygen saturated solution at a scan rate of 5 mV/s. The following equation was used to calibrate the experimental potentials.

$E_{RHE} = E_{Hg/HgO} + 0.059\ pH = 0.098 + 0.05 \times 13.84$ (1)

The Tafel slope is a key parameter to describe the electrocatalytic performance and kinetics of a reaction and it can be expressed by the following equation.

Characterization with AC-HAADF-STEM

The AC-HAADF-STEM images of the samples were captured on a double Cs-corrected FEI Titan Cubed Themis G2 300 Thermal-field emission microscope with a probe Cs-corrector working at 300 kV. A convergence angle of 25 mrad and a collection angle range of 38 to 200 mrad were used for imaging. The convergence semi-angle and collection semi-angle of EELS were 25 and 41 mrad, respectively.

Computational detail

Spin polarized density functional theory (DFT) calculation was performed by using the Vienna Ab initio Simulation Package (VASP) software package. The ion-electron interactions are described with the projector-augmented wave (PAW). The generalized gradient approximation (GGA) in the form of the Perdew, Burke and Ernzerhof (PBE) exchange-correlation functional was employed with Hubbard correction. The adopted U and J values for Ni are 7.9 eV and 1.0 eV, respectively. The performance of oxygen evolution reaction (OER) was studied according to the well-established computational hydrogen electrode (CHE) model. Surface models are constructed based on CuO(111) facet which has been widely discussed as the most stable surface. A vacuum layer of 15 Å was included to avoid the interaction between neighboring images in periodic boundary condition. The convergence threshold in optimization is 0.03 eV/Å in force. The energy cutoff of the plane wave was 450 eV. We use 5×5×1 k-mesh for all calculations of slab models in this work. To consider solvation effect, the hydroxyl (OH*) was stabilized by 0.50 eV, and hydroxyl that is indirectly bound to the surface, i.e., *OOH, was stabilized by 0.25 eV, as suggested by Nørskov, et al.

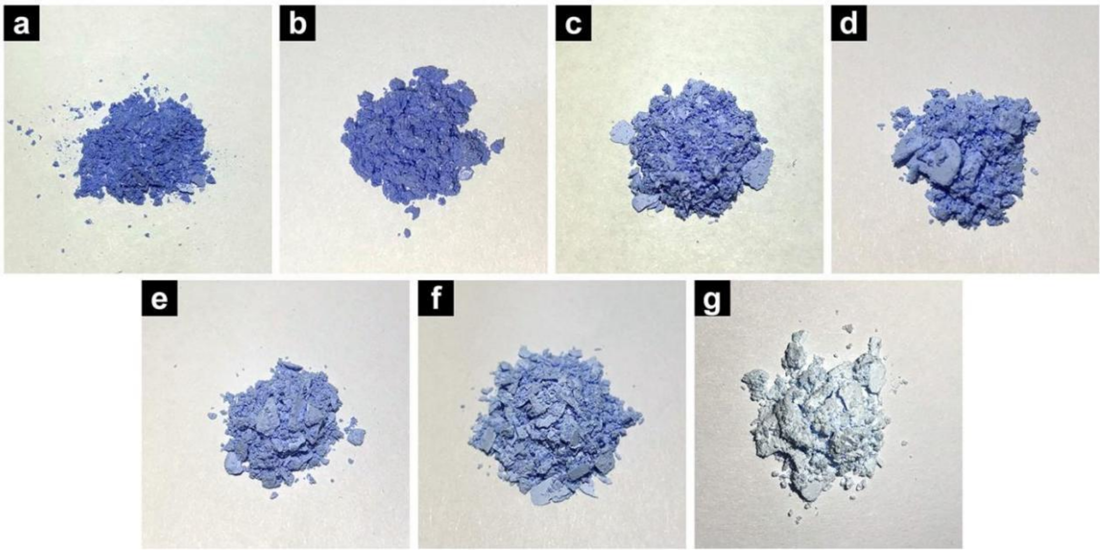

Figure S1. Optical photograph of Cu/Ni bimetallic complexes a-g) P1-P7.

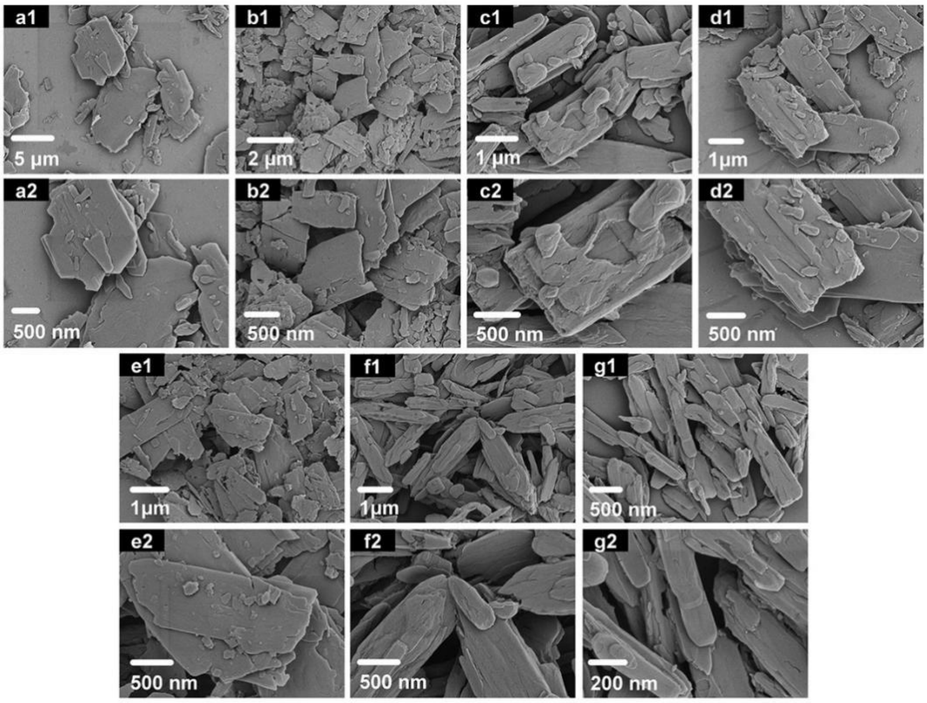

Figure S2 Field emission scanning electron microscopy (FE-SEM) images of Cu/Ni bimetallic complexes a-g) P1-P7.

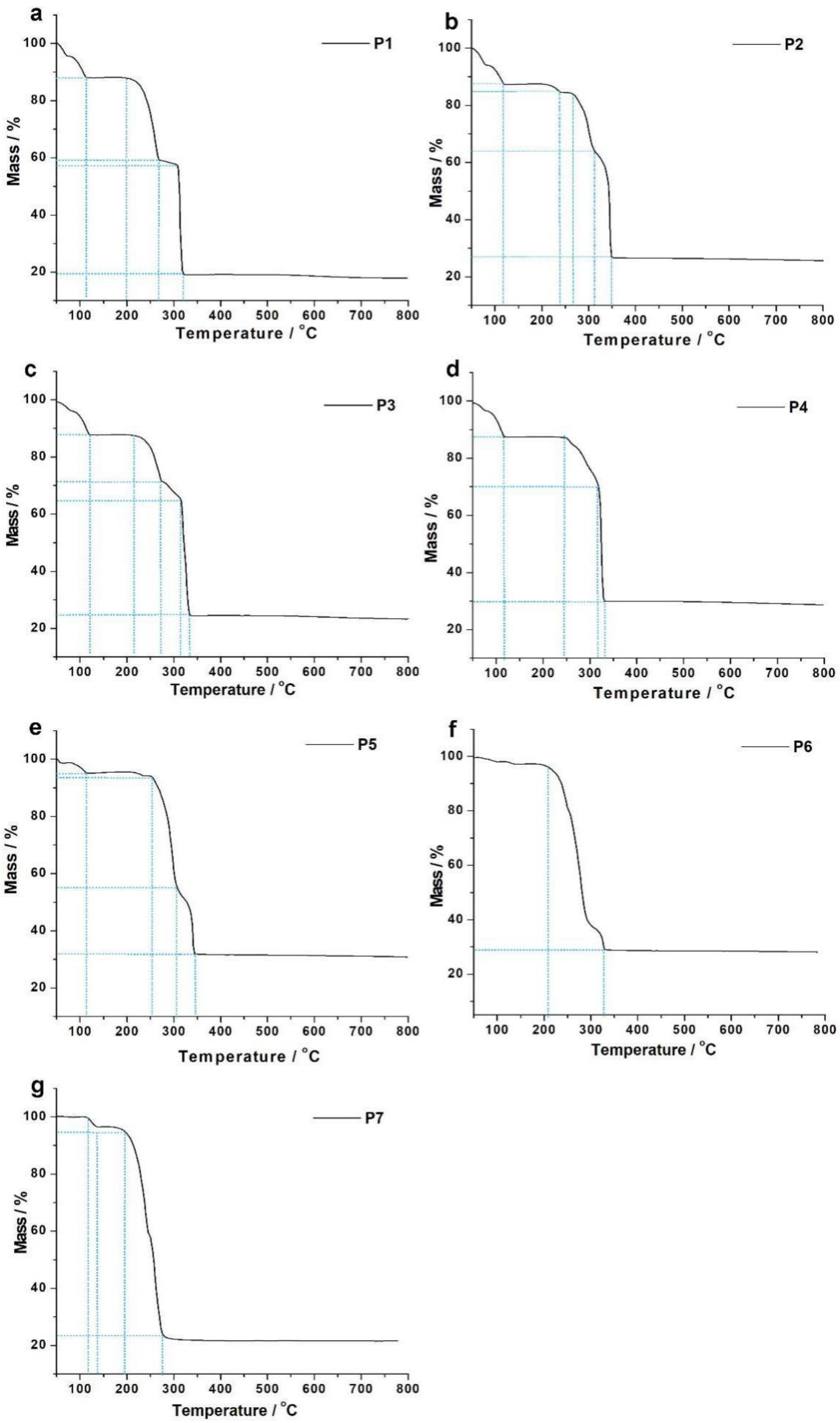

Figure S3 Synchronous thermal analysis curves of Cu/Ni bimetallic complexes a-g) P1-P7.

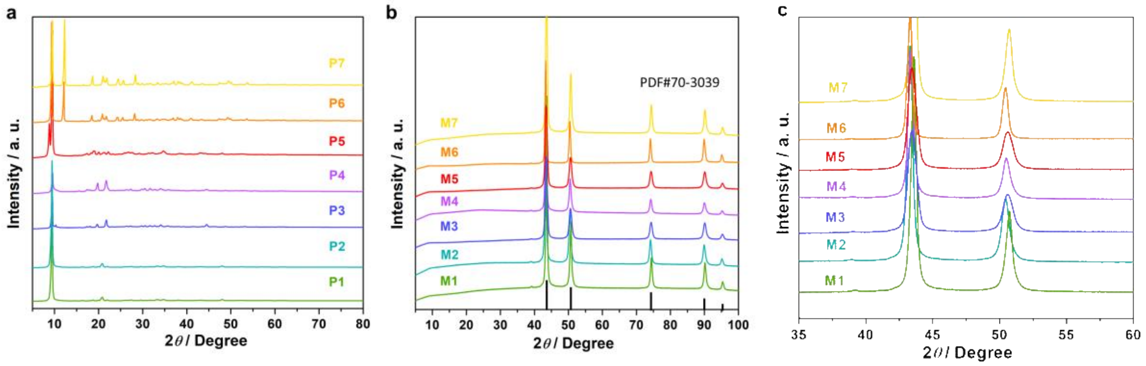

Figure S4 XRD patterns of a) P1-P7, b) M1-M7, and c) enlarged image of b).

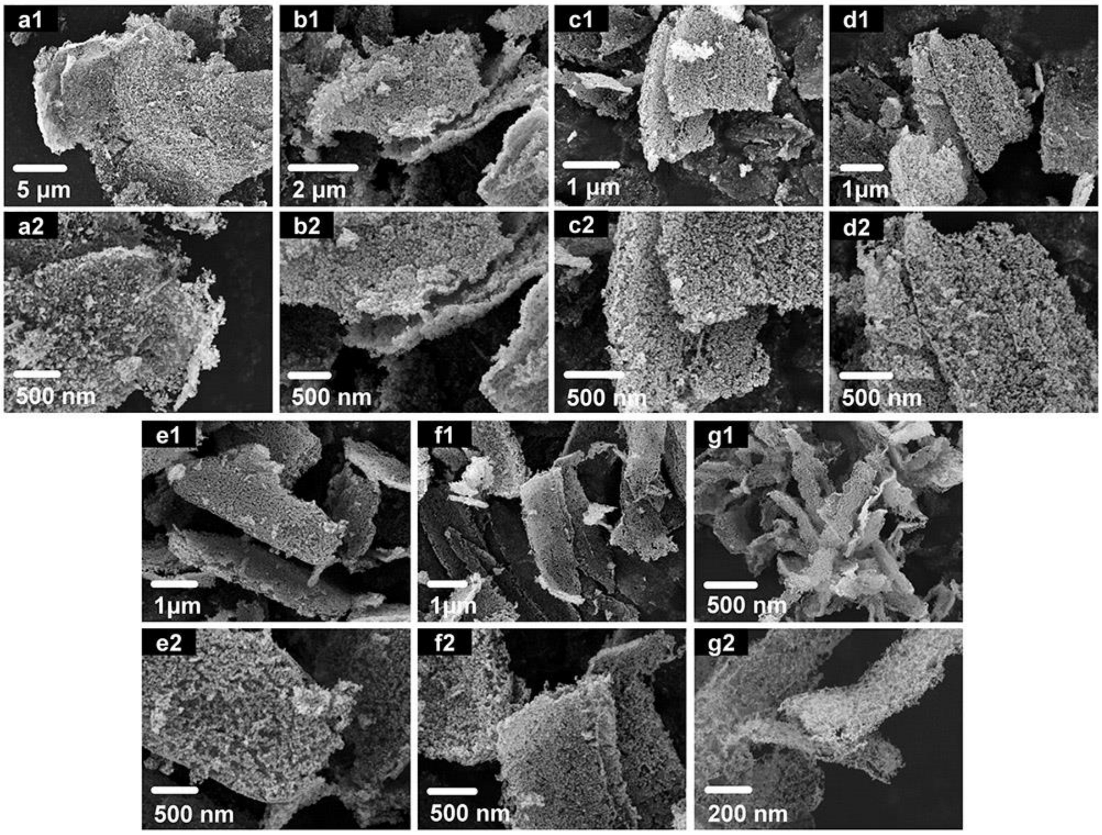

Figure S5 FE-SEM images of CuNi alloy NPs@N/C: a-g) M1-M7.

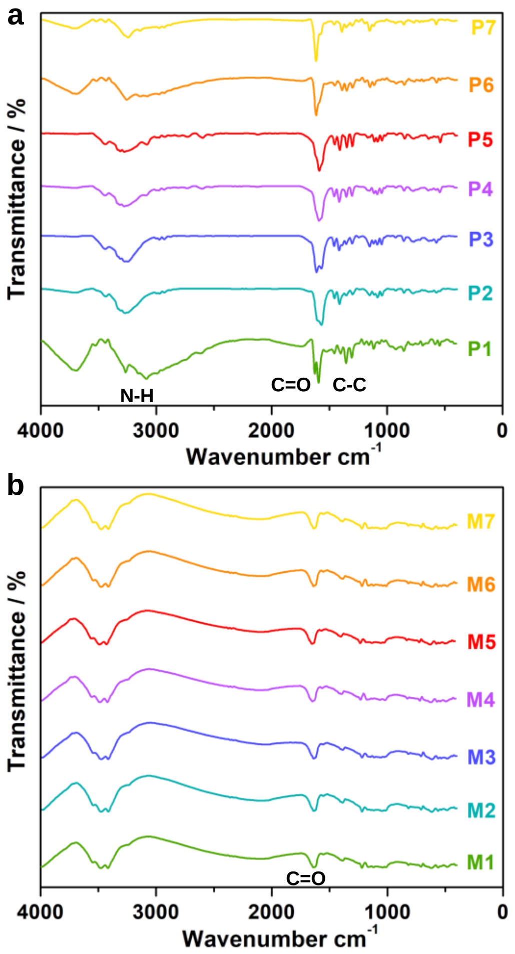

Figure S6 FT-infrared radiation (FT-IR) spectra of as-prepared samples a) P1-P7, b) M1-M7.

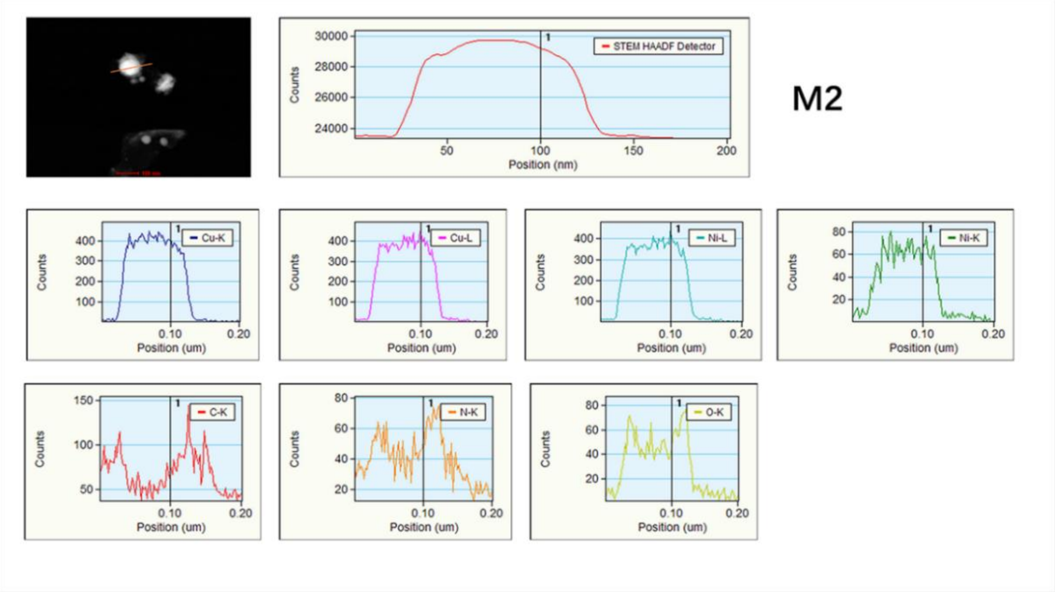

Figure S7 The EDS line scan of M2.

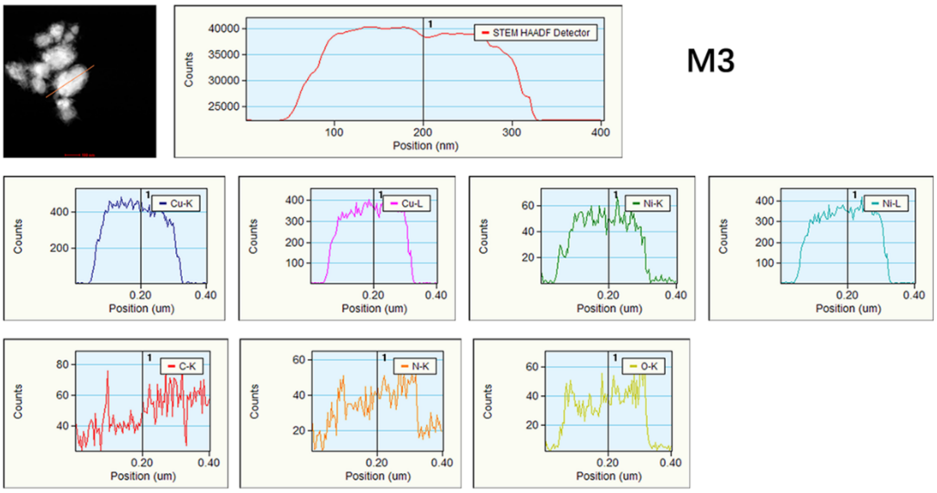

Figure S8 The EDS line scan of M3.

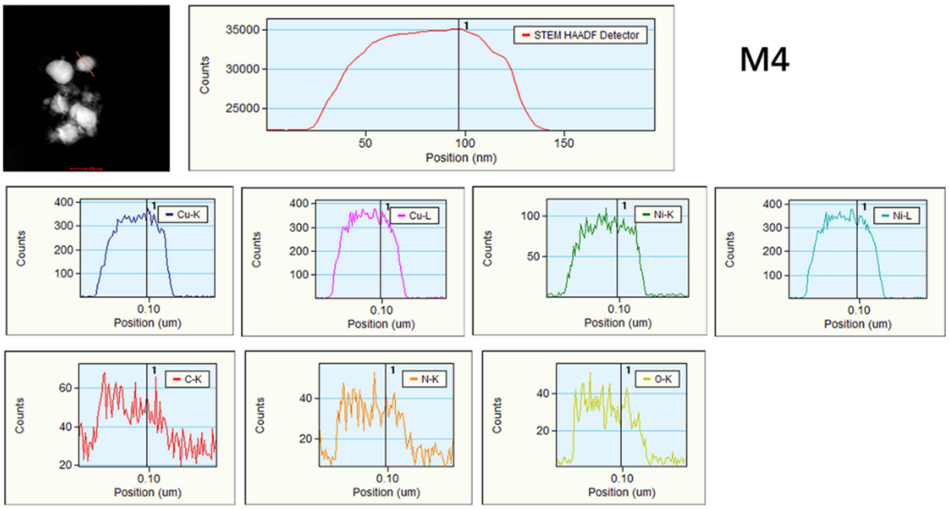

Figure S9 The EDS line scan of M4.

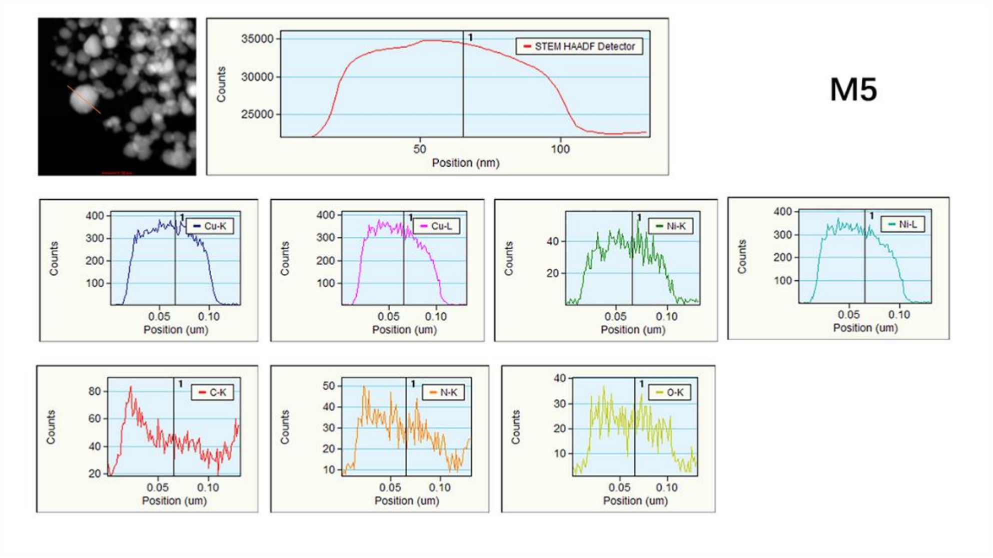

Figure S10 The EDS line scan of M5.

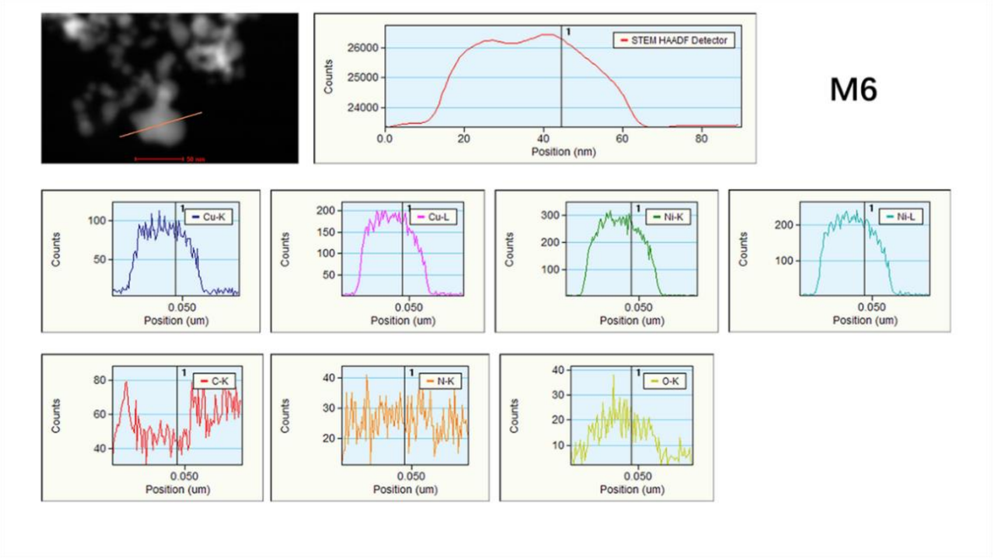

Figure S11 The EDS line scan of M2-M6.

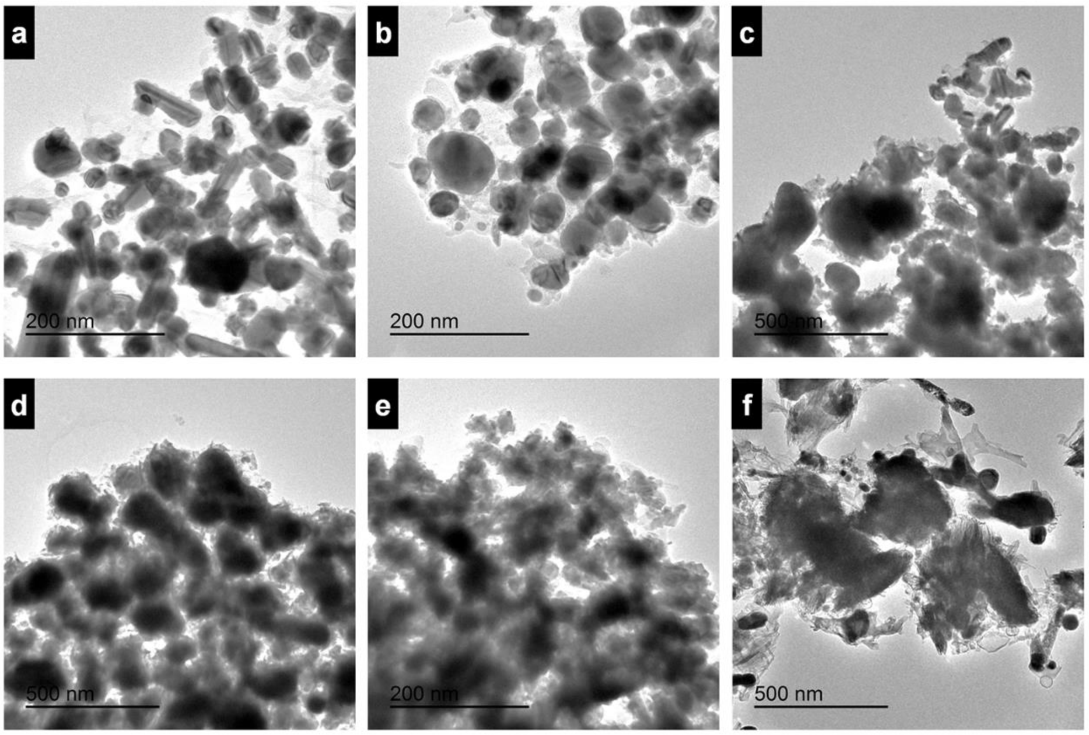

Figure S12 TEM images of M2 after CV test of a-c) 50 cycles and d-f) 100 cycles.

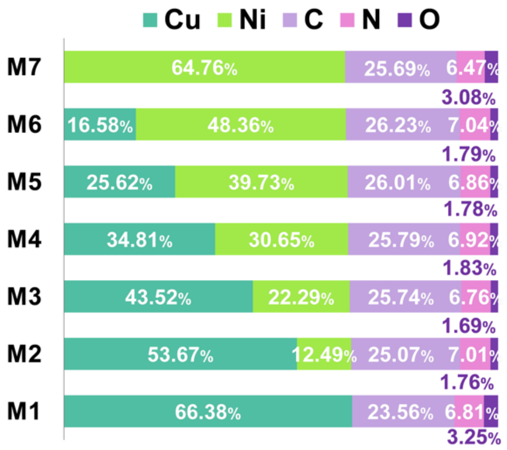

Figure S13 The percentages of Cu, Ni, C, N, and O elements in M1-M7.

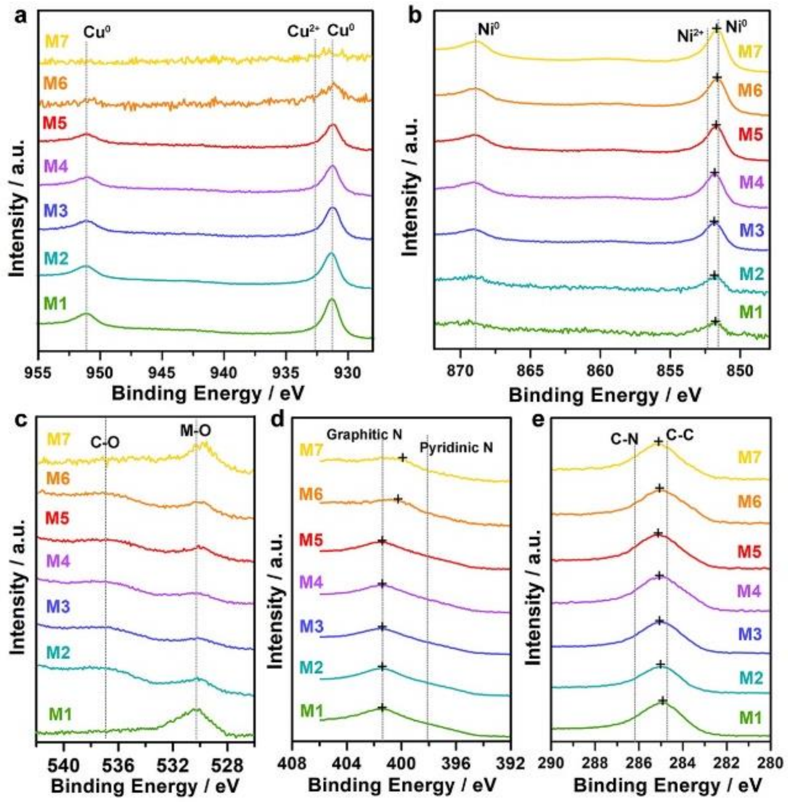

Figure S14 The X-ray photoelectron spectra (XPS) of M1-M7: a) Cu 2p; b) Ni 2P; c) O1s; d) N 1s; e) C 1s.

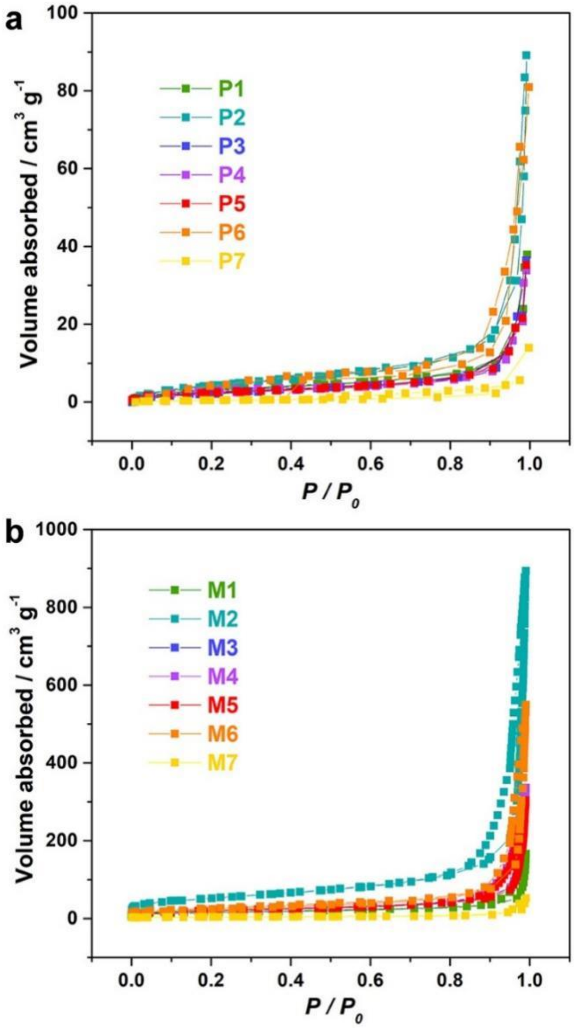

Figure S15 Isothermal nitrogen adsorption desorption curve of a) P1-P7; b) M1-M7.

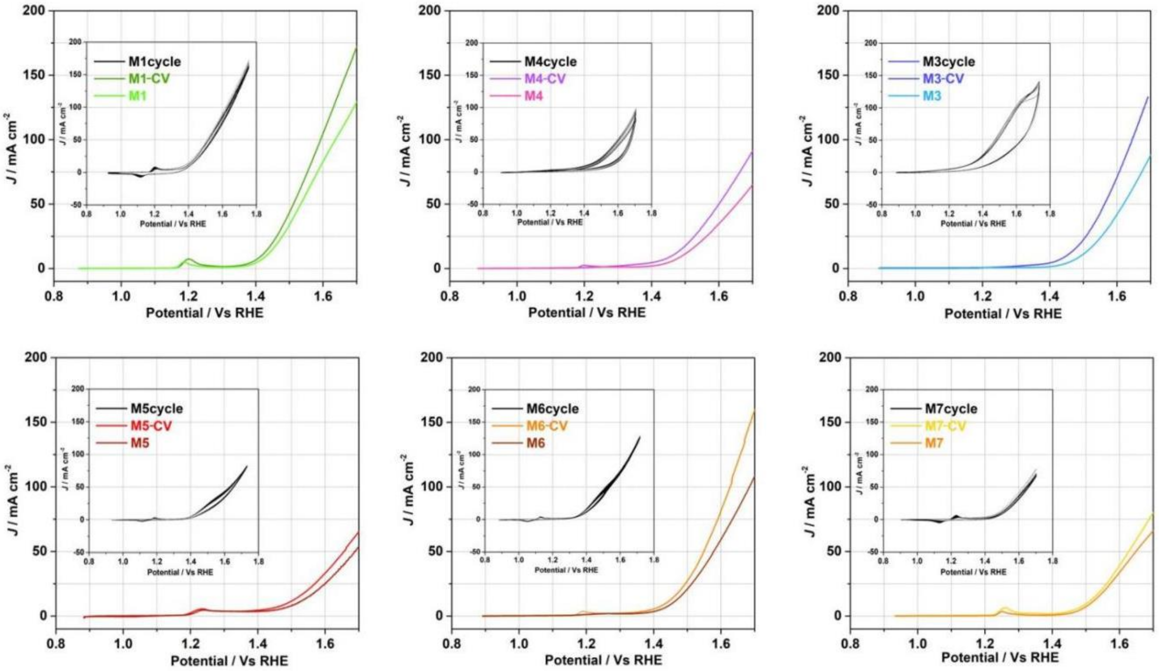

Figure S16 LSV curves before/after electrochemical activation CV of M1 and M3-M7 for 100-cycle.

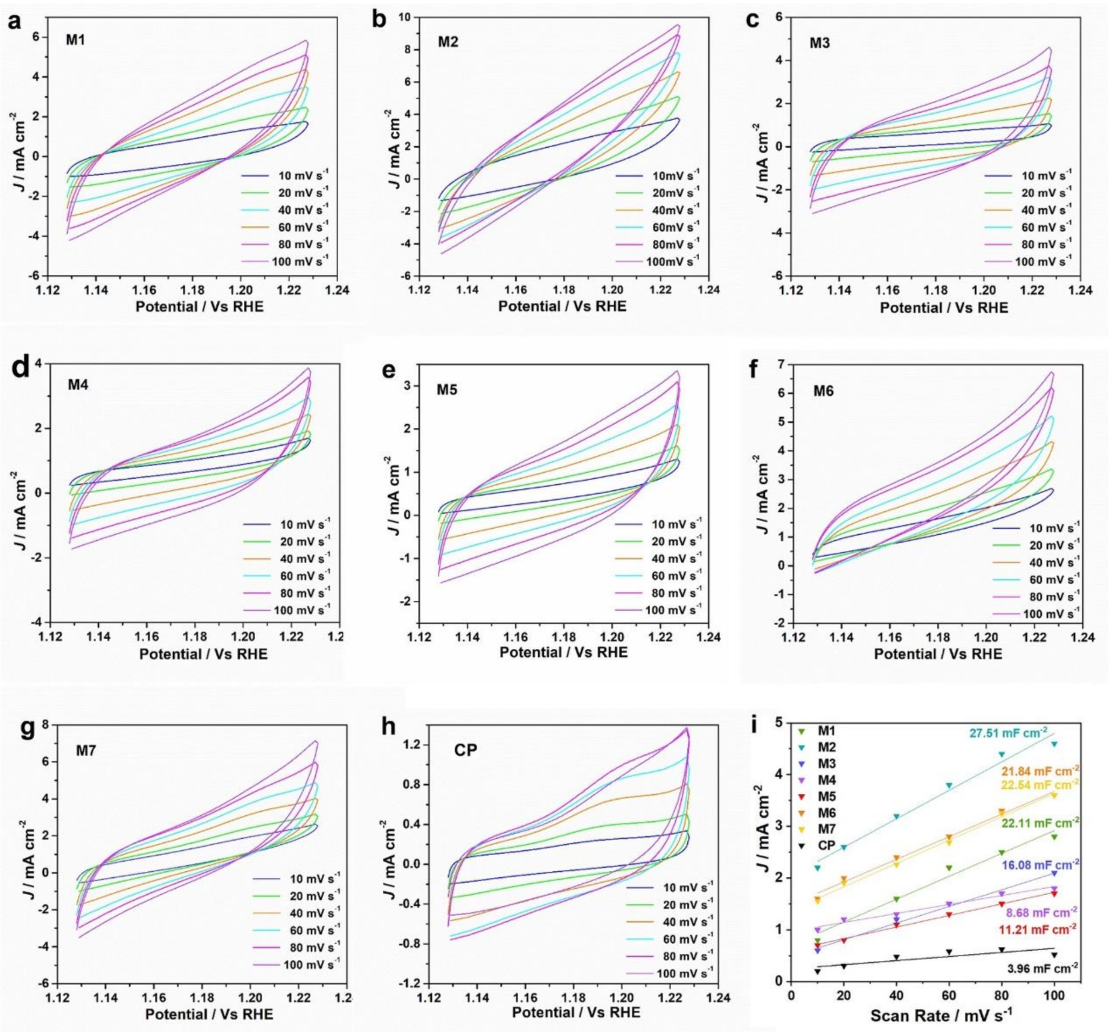

Figure S17 Cyclic voltammograms of M1-M7 after electrochemical activation at scan rates from 10 to 100 mV s^{-1} based on carbon paper: a) M1, b) M2, c) M3, d) M4, e) M5, f) M6, g) M7, h) carbon paper, i) Estimating the C_{dl} and relative electrochemically active surface areas.

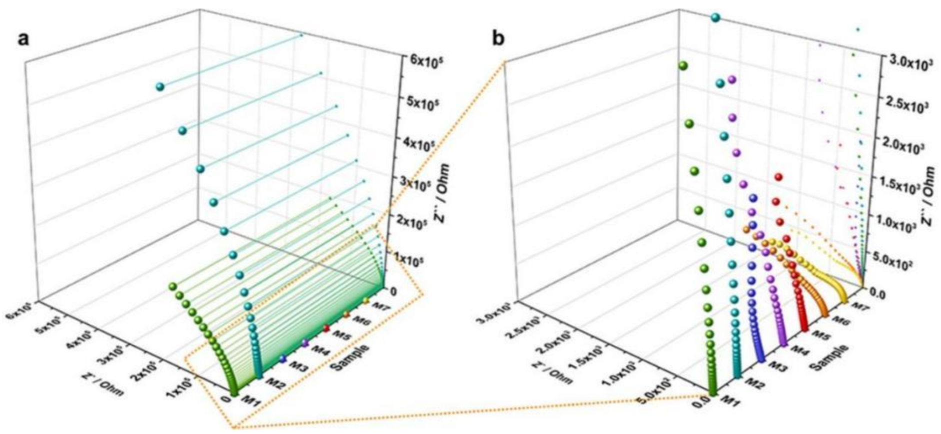

Figure S18 a) Alternating-current (AC) impedance curves of M1-7, b) magnifying image of AC curves.

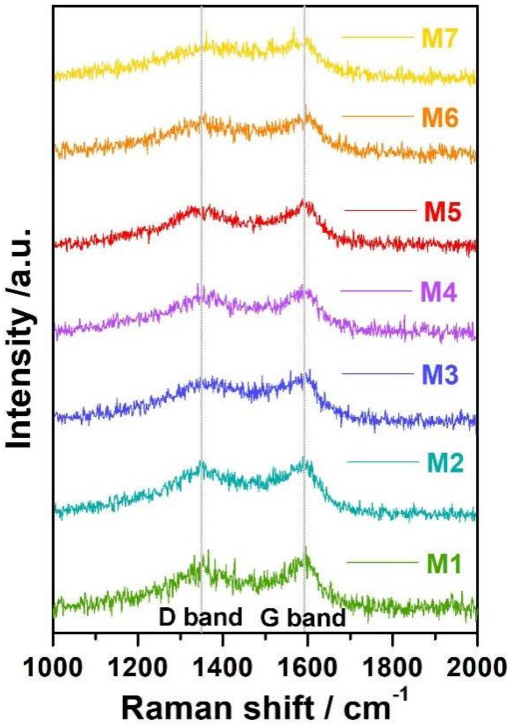

Figure S19 Raman spectra of M1-M7 after CV activation.

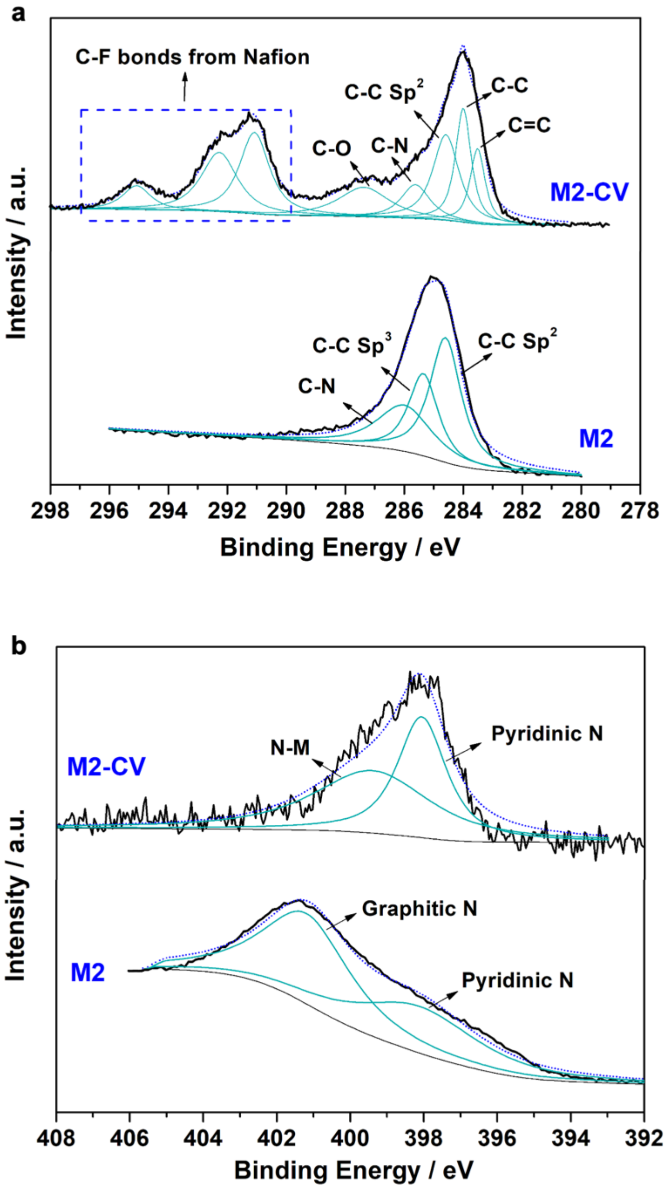

Figure S20 The high-resolution XPS spectrums of M2 and M2 after CV test of 100 cycles noted as M2-CV: a) C1s; b) N 1s.

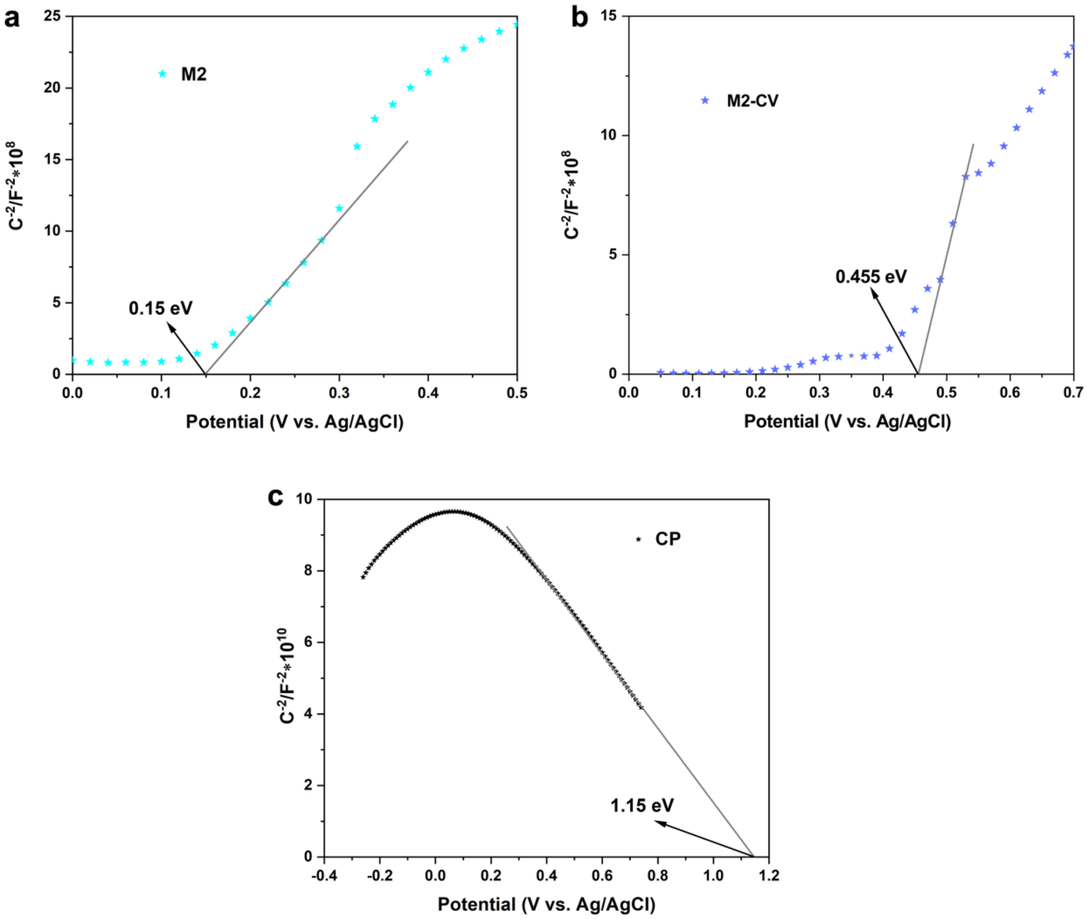

Figure S21 Mott-Schottky curves of a) fresh M2, b) M2-CV, and c) CP.

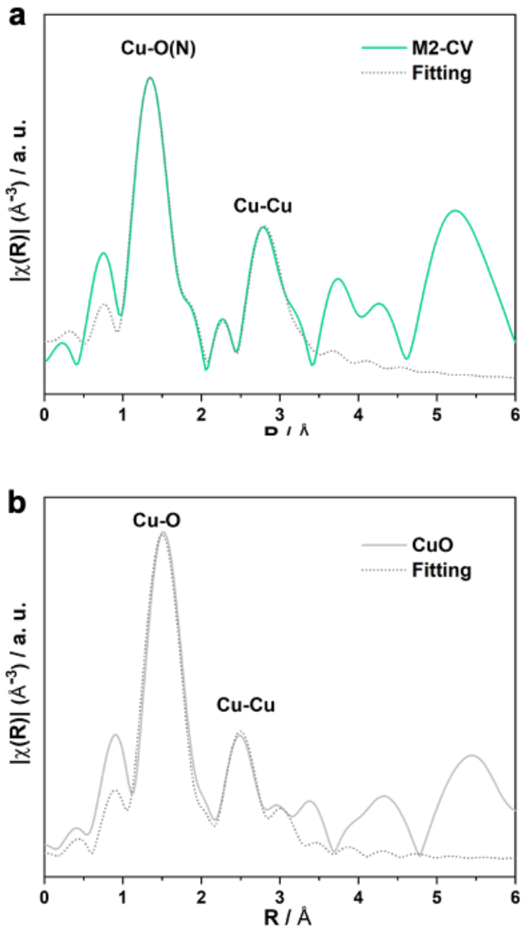

Figure S22 In Cu K-edge k^3-weighted R-space plots of M2-CV.

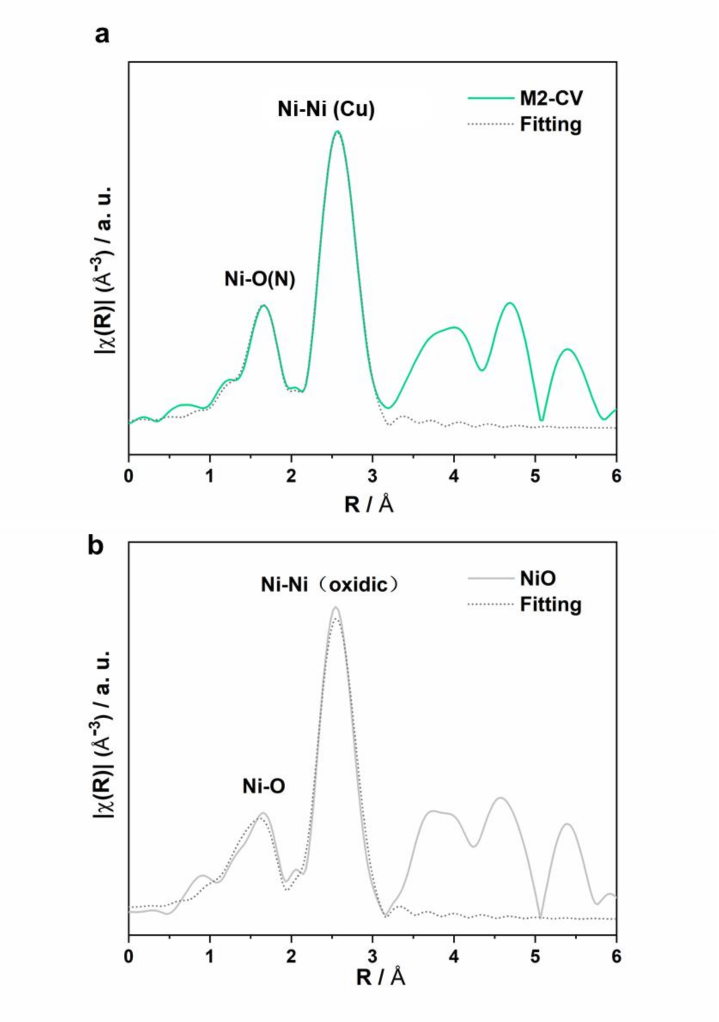

Figure S23 In Ni K-edge k^3-weighted R-space plots of M2-CV.

Sample	Path	C. N.	σ^2*10^3 ($Å^2$)	ΔE (eV)	R (Å)	R_{factor}	S_0^2
CuO	CuO	4*	1.56±0.23	-1.60±2.03	1.94±0.01	0.0099	0.78
	Cu-Cu	4*	1.99±0.71		2.93±0.05		
M2-Cu	Cu-O	2.4	9.47±3.50	1.55±0.43	1.95±0.09	0.0034	
	Cu-N	2.2	9.26±2.75		2.02±0.12		
NiO	Ni-O	6	10.73±3.07	-6.08±0.88	2.09±0.01	0.0077	0.81
	Ni-Ni	12	8.78±1.02		2.96±0.01		
M2-Ni	Ni-N	1.8	6.36±0.01	-2.78±0.63	2.08±0.01	0.0019	
	Ni-O	4.2	8.69±0.01		1.98±0.01		
	Ni-Ni	1.0	2.89±1.27		2.95±0.01		

Table S2 Structural parameters calculated from the EXAFS fittings of the first shell at Cu K-edge and Ni K-edge for M2-CV test as well as CuO and NiO.
